# Cause of death in Chinese Takayasu arteritis patients

**DOI:** 10.1097/MD.0000000000004069

**Published:** 2016-07-08

**Authors:** Jing Li, Mengzhu Zhu, Mengtao Li, Wenjie Zheng, Jiuliang Zhao, Xinping Tian, Xiaofeng Zeng

**Affiliations:** a Department of Rheumatology and Clinical Immunology, Peking Union Medical College Hospital, Peking Union Medical College and Chinese Academy of Medical Sciences, Key Laboratory of Rheumatology and Clinical Immunology, Beijing; b Department of Rheumatology, Chinese Medicine Hospital in Linyi City, Linyi, Shandong, China.

**Keywords:** Causes of death, Risk factors, Takayasu arteritis

## Abstract

To analyze the causes of death and the related risk factors for in-patients with Takayasu arteritis (TAK) admitted to a referral center of China during 1983 to 2014.

The medical charts of 12 deceased TAK patients (10 women, 2 men) were reviewed by two senior rheumatologists. The demographic data, clinical manifestations, angiographic presentations, and the direct causes of death were analyzed retrospectively. Medical records of 40 TAK patients (32 women, 8 men) were selected as controls by age and sex matching method from 81 patients who were sampled isometrically from 810 successively admitted TAK in-patients of the same center during the same period. In addition to the comparison of clinical manifestations between the two groups, binary logistic regression was conducted to explore the related risk factors of mortality of TAK.

Twelve patients died at the median age of 33.5 (ranging from 13 to 68 years old). The median survival time was 102.5(ranging from 6 to 567) months. The direct causes of death were heart failure in 5 (5/12, 41.7%), hemorrhage in 2 (2/12, 16.7%), pulmonary infection in 2 (2/12, 16.7%), sudden death in 1 (1/12, 8.3%), postoperative complication in 1 (1/12, 8.3%), and end-stage malignancy in 1 (1/12, 8.3%). Ischemia (4/12, 33.3%) and hemorrhage (4/12, 33.3%) were the two most common presentations in deceased patients. Eight patients had received surgical procedures related to TAK changes. Among them, 2 patients died after surgical procedure, the other 6 patients died later of non-operation-related causes. Compared with the control group (n = 40), patients in the deceased group had longer disease duration (*P* = 0.017), higher proportion of active disease (*P* = 0.020), secondary hypertension (*P* = 0.004), and congestive heart failure (*P* = 0.017). A model of binary logistic regression had revealed that secondary hypertension (odds ratio [OR] = 9.333, 95% confidence interval [CI]: 1.721 – 50.614, *P* = 0.010), congestive heart failure (OR = 5.667, 95% CI: 1.248 – 25.734, *P* = 0.025), and longer disease duration (OR = 1.007, 95% CI: 1.001 – 0.735, *P* = 0.027) were risk factors for TAK mortality. Active disease (OR = 0.167, 95% CI: 0.038 – 50.614, *P* = 0.018) was negatively associated with death of TAK.

Heart failure is the leading cause of death in TAK patients, followed by ischemia and pulmonary infection. Early deaths occur postoperatively but become rare later after the procedure. Well-control of hypertension, and prevention of congestive heart failure may improve the long-term prognosis.

## 1 Introduction

Takayasu arteritis (TAK) is a rare, chronic, idiopathic systemic vasculitis mainly involves aorta and its major branches, including pulmonary arteries and coronary arteries.^[1]^ The mortality of TAK is generally low although many large arteries are involved. In previous cohort studies of Chinese TAK patients, the common major causes of death were cerebral hemorrhage^[2]^ and congestive heart failure.^[3]^ Coronary arteries involvement was also a major reported cause of sudden death in young TAK patients.^[4,5]^ In long-term follow-up, many TAK patients may develop metabolic syndrome,^[6]^ which could also implicate coronary arteries and lead to cardiovascular diseases that cause death. We reviewed the medical charts of 810 TAK patients admitted to our center over the past 30 years in order to identify the possible related risk factors for death among these patients.

## 2 Patients and methods

### 2.1 Patients

Medical charts of 810 patients fulfilling ACR 1990 TAK criteria^[7]^ were reviewed in this study. These patients were admitted successively to Peking Union Medical College Hospital during 1983 to January 2014. Among them, 12 patients (10 women, 2 men) died. Clinical manifestations of 10 patients (8 women, 2 men) with complete medical records were analyzed for their causes of death, including direct causes, probable causes, and possible causes. Their clinical characteristics were summarized. The causes of death of the other two patients were speculated based on their medical records at death. Eighty-one patients were sampled isometrically from 810 TAK in-patients by SAS software (Version 9.2; SAS Institute, Cary, NC, USA). Medical records of 40 patients (32 women, 8 men) were selected by age and sex matching for the deceased cases. Their demographic data and clinical manifestations were compared with the deceased group.

### 2.2 Methods

Medical records of all patients were retrospectively reviewed. Data including demographic information, disease duration, disease activity status, angiographic presentations, complications,^[8]^ operation and surgical interventions, and causes of death were collected and analyzed. Medical charts of the deceased patients were reviewed by two senior rheumatologists independently. Specific information about the causes of death were available in 10 patients (83.3%). However, the direct causes of death of the other 2 (2/12, 16.7%) patients could not be assessed since they were not stated in the medical records. The causes of death of these two patients were deducted based on the information derived from the medical charts as well as the nursing records. The medical records of the control group were reviewed and summarized simultaneously in the same way. Comparison of clinical manifestations between the two groups was made to investigate whether significant differences existed. Based on the differences, binary logistic regression analysis was used to explore the risk factors of mortality in TAK. The study protocol was approved by Institutional Review Board of Peking Union Medical College Hospital. Because this study was based on a review of medical records that had been obtained for clinical purpose, the requirement for written informed consent was waived.

### 2.3 Statistical analysis

Due to the small sample size, the numerical variables were not distributed normally. We described the numerical variables as median (quartiles) or median (range), and categorical variables as number (percentage). Comparisons between groups were made using Mann–Whitney *U* test for numerical data, and Chi-square tests for categorical data. Fisher's exact tests were performed when the expected frequencies were less than 5. A model of binary logistic regression was adopted to analyze the risk factors for death. Results from logistic regression were expressed as odds ratio (OR) with 95% confidence interval (CI). A two-sided *P* value less than 0.05 was considered to be statistically significant. Analysis was performed with the SPSS software (version 19.0, IBM spss statistics, Armonk, New York, USA).

## 3 Results

In the deceased group, 12 patients (10 women, 2 men) died in our center during 1983 to January 2014. The age at death of these TAK patients ranged from 13 to 68 years old (the median age was 33.5 years old), and their survival time ranged from 6 to 567 months (the median survival duration was 38.5 months). Four patients (4/10, 40%) were at active disease, while 6 patients (6/10, 60%) were at stable disease, and 2 patients with undetermined disease activity. Most of these patients (8/10, 80%) had type V angiographic presentation,^[9]^ and the other two patients (2/10, 20%) had the blood vessels involved limited to the major arteries above the diaphragm (one with type I, and the other one with type IIb). As Ishikawa^[10]^ suggested, these TAK patients were divided into three groups according to the serious complications that could lead to poor prognosis. Six patients (6/10, 60%) were in group III with two or more serious complications, 3 patients in group II (3/10, 30%) and 1 in group I (1/10, 10%), respectively. The most common serious complications were secondary hypertension in 8 patients (8/10, 80%), which included renovascular hypertension and hypertension due to coarctation of the aorta, followed by aortic regurgitation in 4 (4/10, 40%), aortic aneurysm in 2 (2/10, 20%), and retinopathy in 2 (2/10, 20%) (Table 1).

**Table 1:**
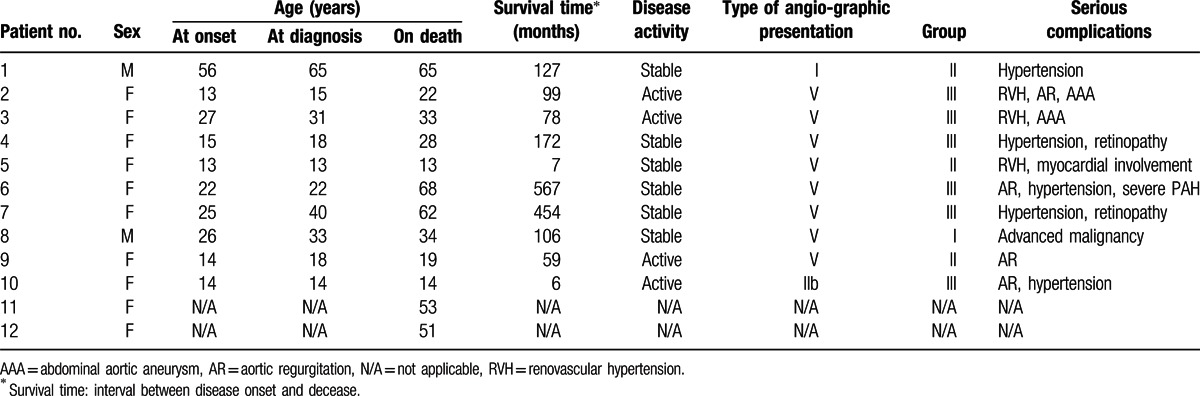
Demographic characteristics, disease activity, type of angiographic presentations, and group of serious complications of TAK patients (n = 12).

The direct cause of death in these TAK patients was heart failure in 4 (4/12, 33.3%), followed by hemorrhage in 2 (2/12, 16.7%), pulmonary infection in 2 (2/12, 16.7%), sudden cardiac death due to severe pulmonary arterial hypertension in 1 (1/12, 8.3%), postoperative complication in 1 (1/12, 8.3%), and end-stage malignancy in 1(1/12, 8.3%). Among the probable and possible related causes of death, ischemia (4/12, 33.3%) and hemorrhage (4/12, 33.3%) were common (Table 2).

**Table 2:**
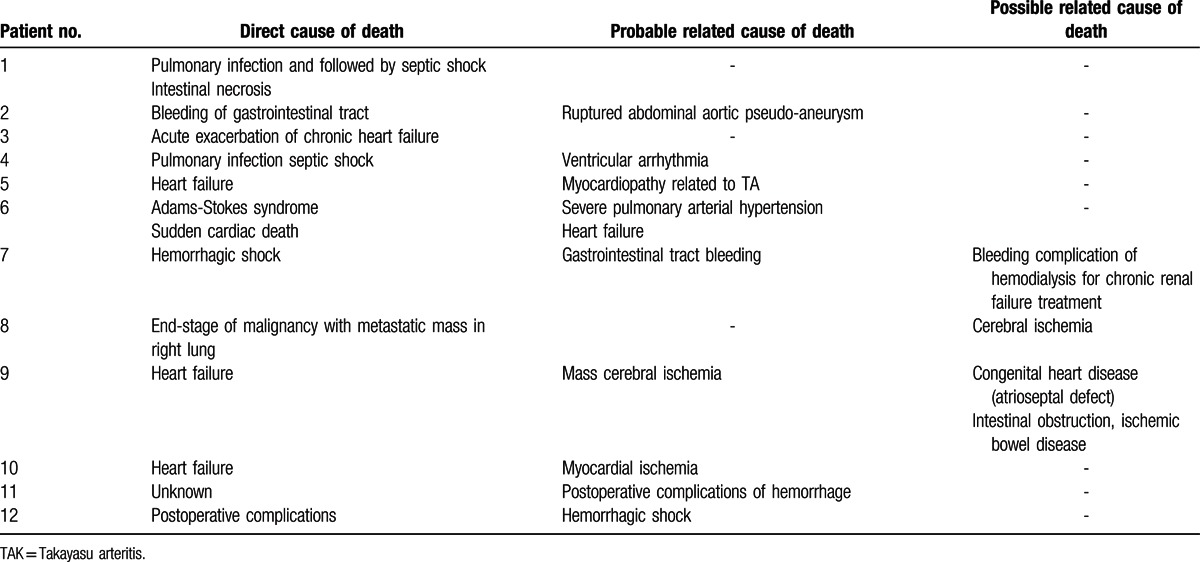
Direct causes, probable related causes, and possible related causes of TAK patients (n = 12).

In this study, eight patients (8/12, 66.7%) had received different types of surgical procedures related to TAK damages. Stenting (4/12, 33.3%) and bypass (4/12, 33.3%) were equally common in these patients. Four patients also received left nephrectomy, aortic valve replacement, fingers amputation, intestinal resection, and anastomasis for Crohn's disease, respectively. Renal failure was found in 3 patients (3/12, 25%) due to hypertension, renal ischemia, and multi-organ failure, respectively. Malignancy (2/12, 16.7%) and Crohn's disease (1/12, 8.3%) were also found to be complicated in 2 patients (Table 3). Two patients died in hospital early after surgical procedures before 2000, and 5 patients died of other reasons (2 heart failure, 2 hemorrhage in gastrointestinal tract, and 1 end-stage malignancy) far later after surgical procedures. One patient died during the operation because of heart failure caused by severe involvement of coronary arteries in 2014 (Tables 2 and 3).

**Table 3:**
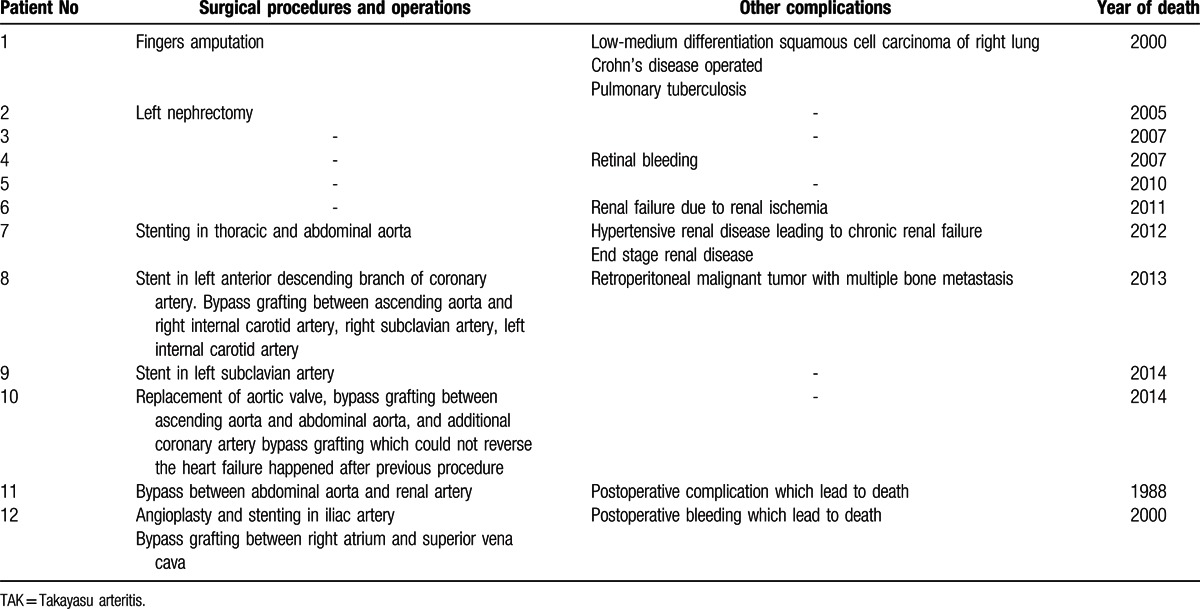
Surgical procedures, other complications, and year of death in 12 TAK patients.

In the control group (32 women and 8 men), the age at onset was 22.0 (16.3, 30.0) years old, age at diagnosis was 25.0 (19.0, 38.5) years, and disease duration from onset to their admission was 36.0 (12.0, 63.0) months. Comparisons of demographic data and clinical manifestations between the deceased group and the control group were made and described in Table 4. There were significant differences in disease duration/survival time (*P* = 0.017), disease activity (*P* = 0.020), incidence of secondary hypertension (*P* = 0.004), and congestive heart failure (*P* = 0.017) between the two groups. No significant difference was found in aortic regurgitation, aneurysm, retinal involvement, pulmonary hypertension, systemic manifestations, nerve system involvement, mucocutaneous lesions, and skeletomuscular system manifestations between the two groups (Table 4).

**Table 4:**
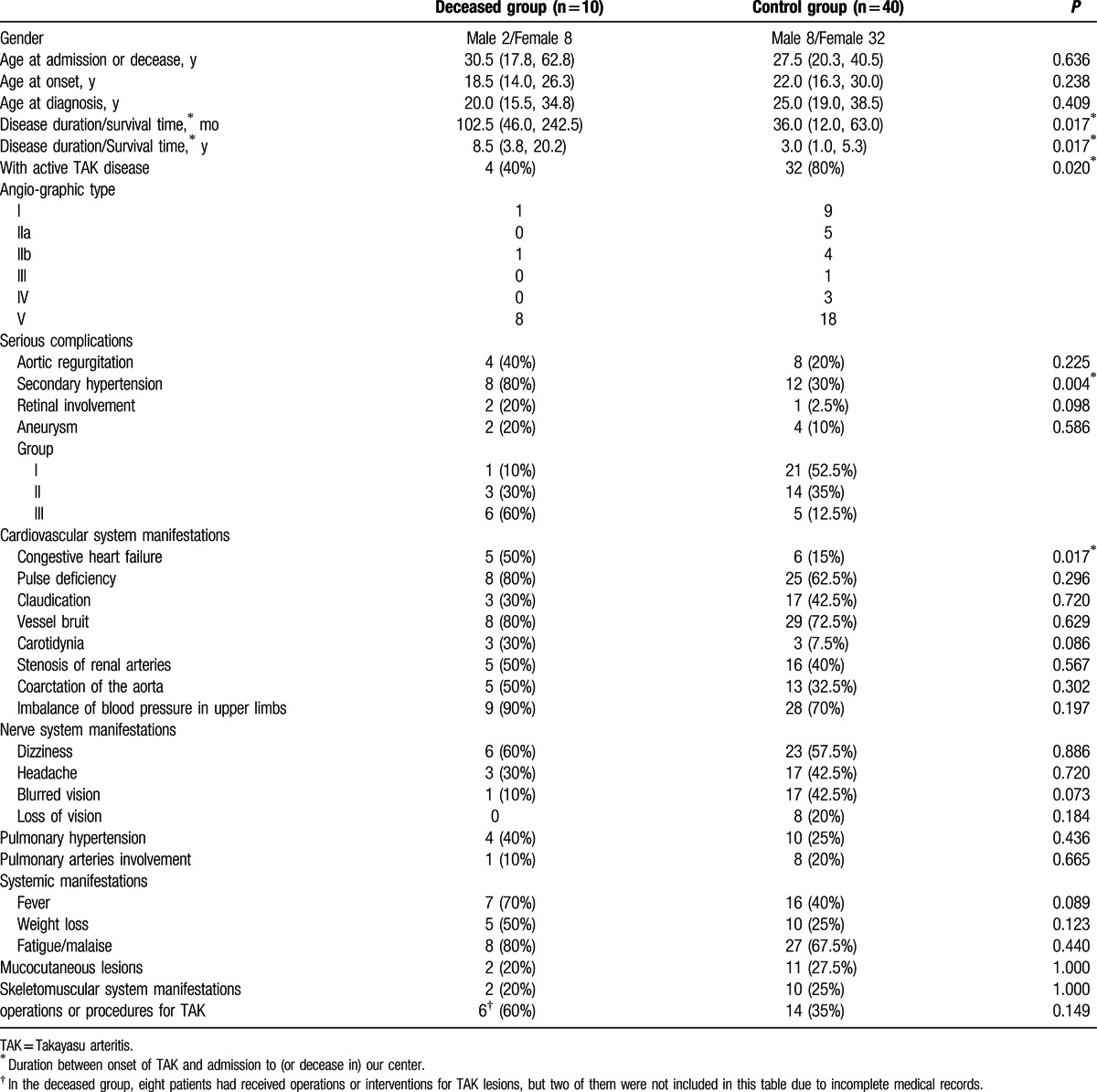
Demographic data and clinical features of deceased group and control group.

Based on the significant differences between the two groups, a model of binary logistic regression was developed to analyze the risk factors for mortality. Differences with *P* value less than 0.05 and between 0.05 and 0.10 were included to test the association between clinical features and death of TAK.

Secondary hypertension (OR = 9.333, 95% CI: 1.721 – 50.614, *P* = 0.010), congestive heart failure (OR = 5.667, 95% CI: 1.248 – 25.734, *P* = 0.025), and longer disease duration (OR = 1.007, 95% CI: 1.001 – 1.014; years, OR = 1.090, 95% CI: 1.010 – 1.177; *P* = 0.027) were identified as risk factors for TAK prognosis. Active disease (OR = 0.167, 95% CI: 0.038 – 50.614, *P* = 0.018), that is, patients who were treated aggressively, was negatively associated with death of TAK. Retinal lesions, carotidynia, blurred vision, and fever, which were not significantly different (*P* = 0.05 – 0.10) in previous Chi-square tests (Table 4), were re-analyzed by the same binary logistic regression model and were found to have no significant association with TAK prognosis (Table 5).

**Table 5:**
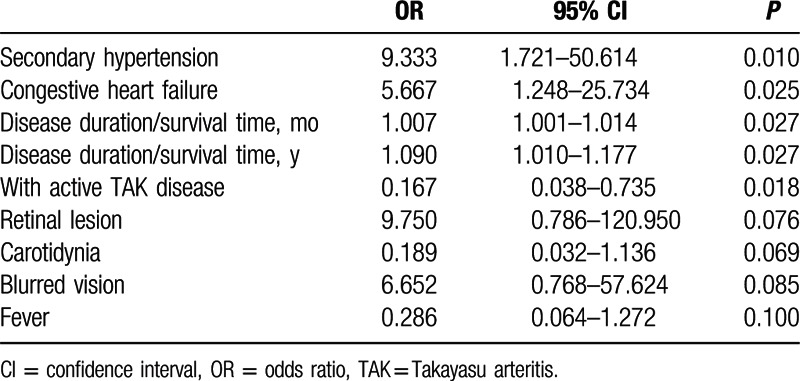
Analysis of risk factors for TAK prognosis by the model of binary logistic regression.

## 4 Discussion

Takayasu arteritis (TAK) is known as a chronic vasculitis, and the disease in many patients develops latently, especially in those with renal artery stenosis or aorta coarctation as the prominent problem. Secondary hypertension may affect patient's heart persistently without evident symptoms, even after irreversible cardiac changes emerged, which in turn lead to heart failure. The most common cardiac manifestation of TAK is aortic regurgitation,^[11]^ which could lead to myocardial remodeling and left ventricle dysfunction, then result in left heart failure. Coronary artery lesions and myocardial involvement are both uncommon in TAK, and have been reported in the literature.^[4,5]^ Combined with aortic regurgitation, retinopathy, aneurysm, secondary hypertension, including renovascular hypertension and hypertension due to coarctation of the aorta, are four serious complications which are considered to be associated with poor prognosis of TAK.^[10]^

A number of case series of TAK had reported the mortality and risk factors for mortality.^[12–18]^ But the low incidence of TAK and the variety criteria adopted in different studies made the comparison between these studies difficult. Just like our study, some of these studies span many decades, and there have been improvement of general medical and surgical care during that time. These changes made the interpretation of data more complicate. Park et al^[17]^ (Table 6) examined the influence of serious complications on survival rates. The presence of two or more serious complications led to higher 5-years (69.9%) and 10-years (36.7%) mortality rates. The serious complications were different in the study of Ishikawa and Maetini,^[12]^ which included valvular heart disease, stroke, heart failure, retinopathy, and renovascular hypertension. However, clinical manifestations, initial disease activity, angiographic classification, laboratory findings, presence and frequency of relapses were not associated with mortality. Ishikawa and Maetini^[12]^ found that the survival varied depending not only on the time of diagnosis, but also the presence of major complications (including retinal microaneurysm formation, hypertension, aortic regurgitation, and aortic or arterial aneurysm), or a progressive course (increasingly symptomatic). They also demonstrated that erythrocyte sedimentation rate (ESR) lower than 20 mm/hr might predict poorer prognosis, which might due to inadequate treatment. Because historically ESR has been regarded as the indicator of active disease, so many physicians treat TAK patients based on the level of ESR. Therefore, for patients with low ESR levels, they might not be treated aggressively so the uncontrolled insidious inflammatory process causes damage in a long-term. In this study, we have also demonstrated that heart failure, secondary hypertension, and disease duration were risk factors of TAK mortality, which in accordance with former cohort studies respectively, by method of case-control study and model of binary regression. In addition, activity of TAK disease was proven to be negatively associated to poor prognosis, which might be similar to the meaning of ESR in Ishikawa and Maetini study.^[12]^ In other words, TAK patients with higher disease activity were more likely to receive aggressive treatment, which may lead to longer survival time.

**Table 6:**
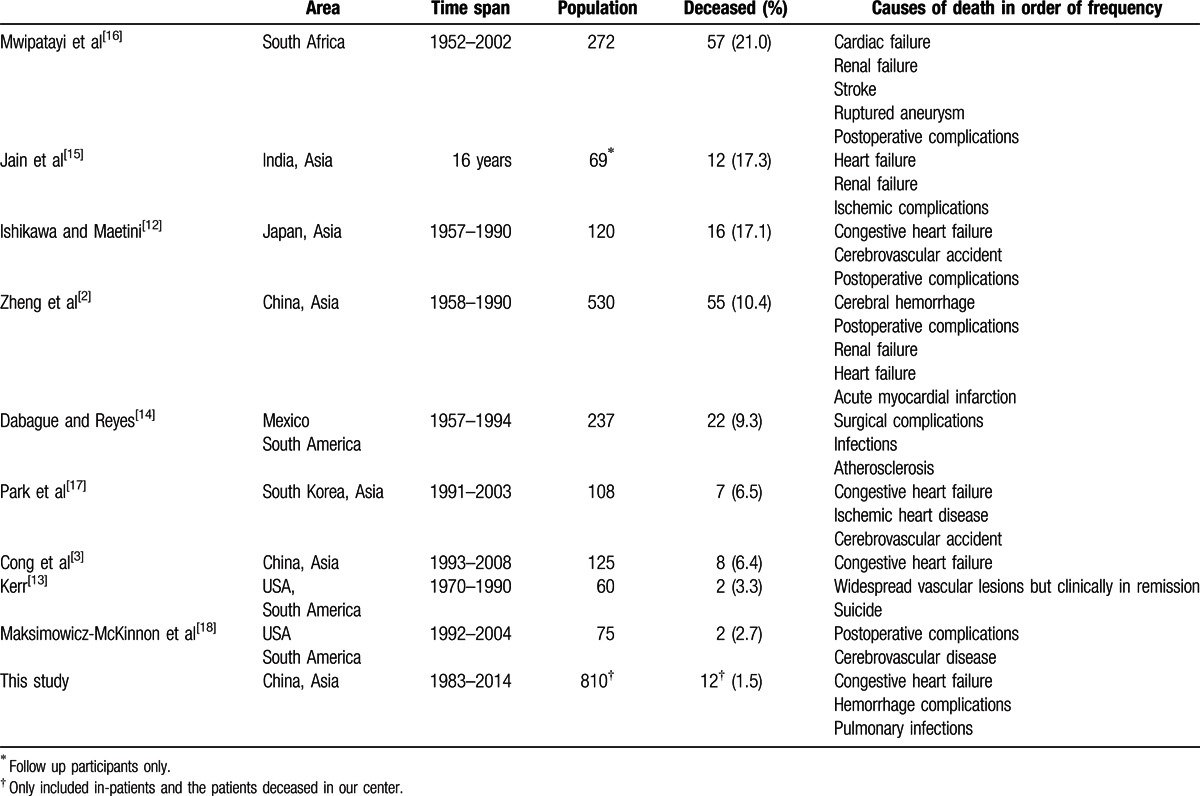
Reported mortality rate and causes of death (listed in order of the mortality rate descending).

In previous cohort studies of Chinese TAK patients, cerebral hemorrhage^[2]^ and congestive heart failure^[3]^ were found to be the main causes of death. Zheng et al^[2]^ discovered that cerebral hemorrhage (23.6%) were more often happened in patients with renovascular hypertension and ischemic complications, which may also be related to the usage of antiplatelet drugs or anticoagulant drugs. In Cong et al study, chronic heart failure was found to be the main cause of death in 5 patients (62.5%).^[3]^ The difference between these 2 studies might be related to the time span of the studies. Zheng's study was reported in early 1990s, and Cong's was in 2010. In recent 20 years, the treatment of TAK, including medication therapy and surgical procedures, has progressed dramatically in China. So the incidence of cerebrovascular disease and heart attack has reduced significantly comparing with the data of TAK cohort 20 years before.

Our study included TAK patients who died in PUMCH over 30 years, mostly in recent 15 years (11/12, 91.7%). The main direct cause of death in our study was heart failure (4/12, 33.3%), followed by hemorrhagic shock (2/12, 16.7%) due to gastrointestinal tract bleeding and pulmonary infection (2/12, 16.7%). The heart failure might be related to the systemic hypertension and aortic regurgitation, and the time for intervention is very critical. Heart failure due to aortic regurgitation with enlarged left ventricle, combined with occlusion of right coronary artery presented with regional myocardial motion abnormality might increase the surgical risk dramatically, as patient 10 in our study. The hemorrhagic complications might be related to the widely use of low-dose aspirin (75 or 100 mg per day), and de Souza et al^[19]^ had proven that antiplatelet therapy was associated with a lower frequency of ischemic events in patients with TAK. So, the widely use of anti-platelet therapy in TAK patients is more beneficial. Pulmonary infection in the men elder patient was secondary to his low-to-medium differentiated squamous cell lung carcinoma. In the young women patient who died of pulmonary infection, the level of her immunoglobulin decreased generally, which might be caused by the rapid progress of infection.

Pulmonary arteries involvement in TAK may include both proximal and distal vessels.^[20,21]^ The main clinical manifestations are caused by the imbalance between ventilation and blood flow of pulmonary circulation.^[22]^ Pulmonary arteries involvement in TAK was not rare, when respiratory symptoms occurred such as dyspnea on exertion, or non-productive cough, investigation for pulmonary hypertension and pulmonary vessel involvement should be initiated. In this study, one patient (1/12, 8.3%) with pulmonary hypertension died from sudden cardiac death.^[23,24]^

Coronary artery involvement was frequently reported as a cause of death for young TAK patients,^[5]^ even in infants.^[4]^ In these patients, death was usually due to myocardial ischemia. In our study, a young woman with right coronary artery occlusion and severe aortic insufficiency died during her operation for aortic valve replacement. Bypass procedures were performed before between her aortic arch and right carotid artery, and between her ascending aorta and abdominal aorta. Despite of those procedures, she still died during the operation due to severe heart failure, which was the reason for her operation. In an observational Brazilian cohort study, the prevalence of metabolic syndrome (MetS) in TAK patients was higher compared with matched healthy control group.^[6]^ TAK patients had higher frequency of hypertension compared with healthy controls, in addition to other characteristics of MetS.^[6]^ Metabolic syndrome has been widely accepted as a risk factor for cardiovascular diseases,^[25,26]^ and development of MetS in TAK might be related to overweight/obesity and higher Framingham score.^[6]^ No correlation was revealed between the development of MetS and age at disease onset, duration of disease, hypertension, current or cumulative prednisone dose, and previous use of immunosuppressive drugs.^[6]^ MetS related cardiovascular lesions in TAK needs further study.

Renal failure was found to be the cause of death in studies of Mwipatayi et al,^[16]^ Jain et al,^[15]^ and Zheng et al.^[2]^ In this study, chronic renal failure happened in patient 7, due to persistent hypertensive renal disease, in spite of stenting in thoracic and abdominal aorta. Patient 6 also developed renal failure due to renal ischemia. So, renal failure was not uncommon in TAK patients, but substantial development in renal replacement therapy could reduce its risk for TAK patients.

It sounds reasonable that extensive involvement in TAK could lead to poorer prognosis, but this speculation has not been verified in clinical studies. In this study, 7 patients (7/10, 70%) had type V angiographic presentation. Another Chinese center for cardiovascular disease reported a retrospective cohort study of TAK and found no association between poor prognosis and angiographic types.^[27]^ In Park et al^[17]^ study, angiographic type was not associated with prognosis neither. However, serious complications, especially hypertension, aneurysm, retinopathy, and aortic regurgitation, were able to predict poor outcome.^[2,3,10,12,17,27]^

Postoperative complications were not rare and might contribute to mortality in TAK patients. However, death caused by surgical procedures has been decreasing. Miyata et al^[28]^ reported 12 patients in a consecutive cohort of TAK patients died in hospital after surgical procedures. But these patients were all died in early decades, no patients died in early stage postoperatively after 1980 in that center. In studies of Zheng et al,^[2]^ Ishikawa and Maetini,^[12]^ Mwipatayi et al,^[16]^ , and Maksimowicz-McKinnon et al,^[18]^ postoperative complications were also the main causes of death. In our center, the situation was similar. Two of 27 consecutive TAK patients died in hospital after surgical procedure before 2000, compared with 1 in 117 consecutive TAK patients died during the operation due to severe heart failure. The dramatic decrease of postoperative mortality in early stage of TAK patients may due to the development of preoperative evaluation, management of surgical risks, and understanding the pathophysiology of arterial reconstruction.

The major limitation of this study is the relatively small sample size. Since the mortality of TAK is low and it takes time for patients to develop serious complications, this study still can provide valuable information for clinicians who are taking care of patients with this rare condition in certain extent. Another limitation is the retrospective nature of this study. The causes of death of 2 patients are deducted based on medical records. Therefore, prospective, long-term follow up studies are needed to verify the risk factors revealed in this study.

## 5 Conclusions

Though cross-sectional study of causes of death in in-patients who died in hospital could not represent the overall causes of death in TAK patients, our study has found that heart failure caused by secondary systemic hypertension and aortic regurgitation are the leading causes of death in TAK. These results are in accordance with other TAK cohort studies. In addition to heart failure, ischemia and hemorrhage are also common causes of death in TAK. Infection, especially pulmonary infection, also threatens the survival of TAK patients. Physicians should be cautious to infection. Early postoperative death has become rare in many referral centers. The right time for surgical intervention still needs further investigation.
